# Shock-Related Thyroid Changes: A Rare Presentation in a Young Patient With Hemorrhagic Shock Secondary to a Road Traffic Accident

**DOI:** 10.7759/cureus.95764

**Published:** 2025-10-30

**Authors:** Ajit Thakur, Prashant Bhatia, Avinash Sharma, Sashank Mavuduru, Anshuman Panda

**Affiliations:** 1 Critical Care Medicine, Asian Institute of Medical Sciences, Faridabad, IND

**Keywords:** hemorrhagic shock, shock thyroid, thyroid edema, thyroid injury, trauma imaging

## Abstract

Shock-related thyroid changes are a rare radiological finding, considered part of the hypovolemic shock complex. It is characterized by thyroidal and perithyroidal edema without direct thyroid injury, typically seen in severe trauma. Very few cases have been described in the literature. We report a case of a 15-year-old male who presented in hemorrhagic shock following a road traffic accident. Whole-body computed tomography (CT) imaging demonstrated heterogeneous thyroid enhancement with perithyroidal edema, consistent with shock-related thyroid changes, in the absence of direct thyroid trauma. The patient also sustained a high-grade splenic injury requiring emergent laparotomy and splenectomy, along with orthopedic intervention for a femur fracture. Thyroid function tests were normal, and the patient gradually improved with appropriate surgical and supportive management. The radiological features of shock-related thyroid changes are distinct and must be differentiated from direct thyroid injury or adjacent vascular damage. The underlying pathophysiology is uncertain, but proposed mechanisms include third-spacing of resuscitative fluids or thyroidal hypoperfusion during profound hypovolemia. Recognition of this entity is crucial as it is self-limiting and does not require specific thyroid-directed intervention. Shock-related thyroid changes are a rare but important secondary imaging finding in the setting of severe trauma and hypovolemic shock. Awareness of this condition can help avoid misdiagnosis and unnecessary investigations.

## Introduction

Shock-related thyroid changes are an uncommon computed tomography (CT) finding that forms part of the hypovolemic shock complex [1]. It was first described by Brochert and Rafoth as diffuse thyroidal and perithyroidal edema without evidence of direct thyroid injury [1]. The condition typically occurs in patients with severe hemorrhagic or hypovolemic shock, often secondary to major thoracoabdominal trauma. Although only a limited number of cases have been reported, awareness of this entity is crucial as it can mimic direct thyroid injury or vascular trauma [2-4]. Direct traumatic thyroid injuries are themselves exceedingly rare, with isolated case reports describing gland rupture, hematoma, or parenchymal disruption following blunt neck trauma [5-7]. Distinguishing these entities from shock-related thyroid changes on imaging is important, as the latter represent a transient physiological response rather than a structural injury [4]. The underlying mechanism remains uncertain but is thought to involve thyroidal hypoperfusion, interstitial fluid extravasation, or a transient hyperdynamic response to profound hypotension [1-4]. Recognizing this self-limiting radiologic finding can prevent misdiagnosis and unnecessary surgical intervention [4]. Shock-related thyroid changes estimated to occur in less than 0.5% of trauma CT scans, whereas direct thyroid rupture accounts for <1% of neck injuries. This case aimed to highlight the radiologic distinction between the two entities for early recognition [1].

## Case presentation

A 15-year-old male was brought to the emergency department following a road traffic accident while driving a two-wheeler. On presentation, he was in hemorrhagic shock with blood pressure not recordable, tachycardia (pulse rate 134 beats per minute), and tachypnea. He had multiple lacerations and abrasions on the elbow, chin, knee, thigh, and foot. The patient was managed in the intensive care unit, where he was investigated and hemodynamically stabilized.

**Figure 1:**
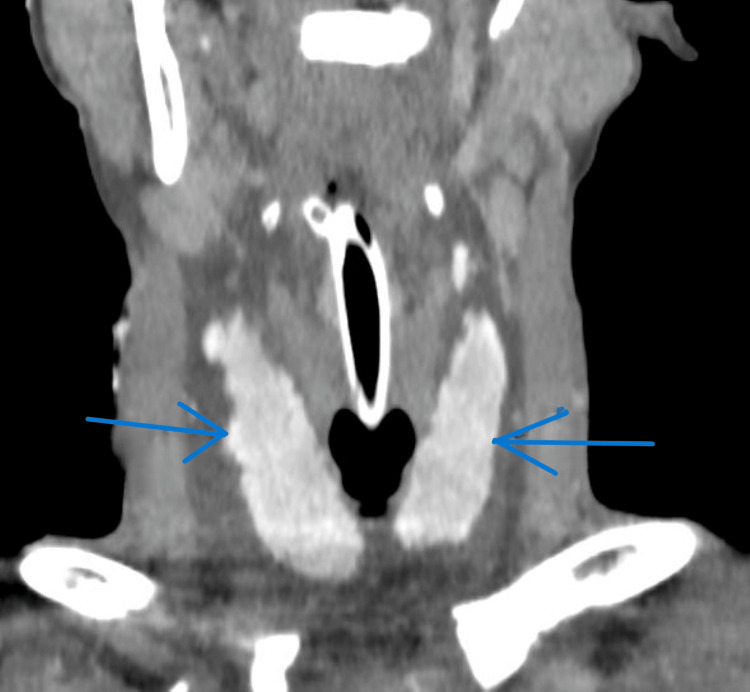
Coronal contrast-enhanced CT of the neck showing shock-related thyroid change. Contrast-enhanced computed tomography (CT) scan of the neck in coronal view (venous phase; slice thickness 5 mm; window/level 350/50 HU) showing diffuse symmetric enlargement of the thyroid gland with perithyroidal low-density fluid (blue arrows) consistent with shock-related thyroid change. The thyroid (T) gland demonstrates intact margins and homogeneous enhancement with irregular contours but without evidence of laceration or hematoma. HU: Hounsfield unit

**Figure 2:**
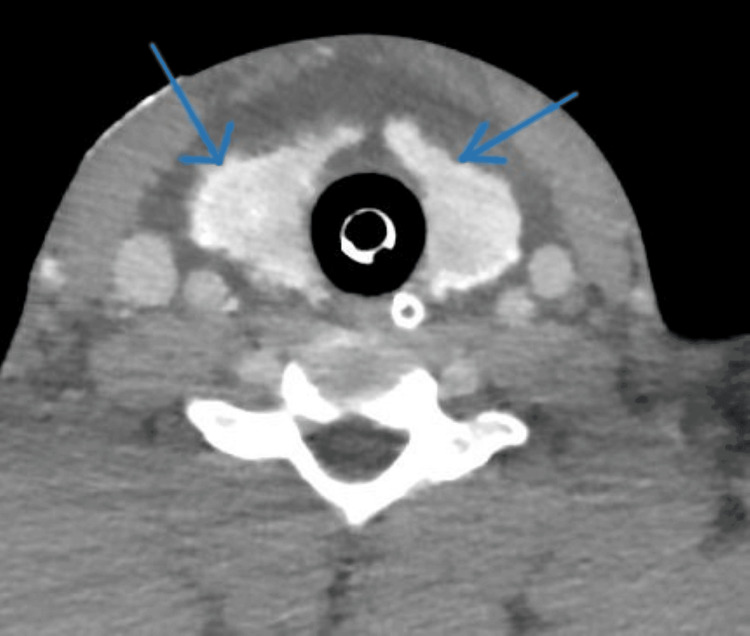
Axial contrast-enhanced CT of the neck demonstrating shock-related thyroid change. Contrast-enhanced CT scan of the neck in axial view (venous phase; slice thickness 5 mm; window/level 350/50 HU) demonstrating bilateral perithyroidal edema and simple fluid (blue arrows) surrounding a normally enhancing thyroid gland, characteristic of shock-related thyroid change. The trachea (Tr) and strap muscles (SM) are visualized for orientation. HU: Hounsfield unit

Whole-body computed tomography (CT) was performed according to the trauma protocol. CT of the head and cervical spine revealed no abnormalities. Contrast-enhanced CT of the neck demonstrated diffuse fascial-plane edema with a low-density collection in the anterolateral neck compartment. Both thyroid lobes appeared homogenous with irregular outlines and intense enhancement, along with perithyroidal fluid and a break in the midline isthmus, suggestive of a shock-related thyroid change (Figures 1, 2).

**Figure 3:**
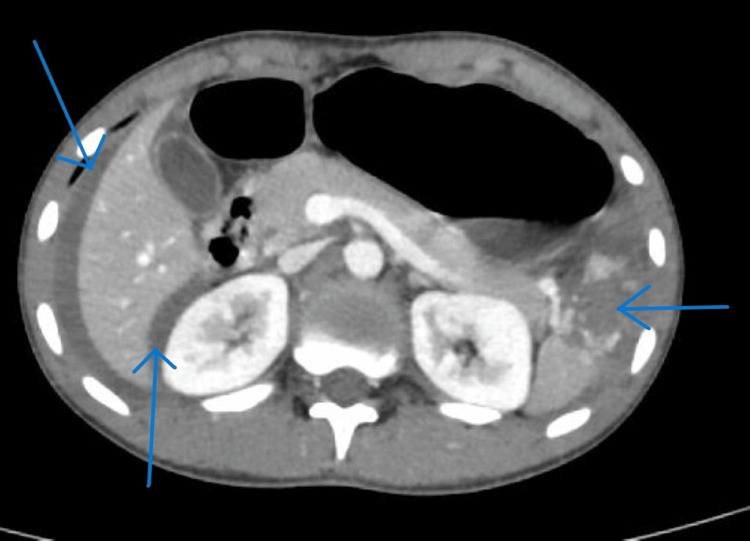
Contrast-enhanced CT of the abdomen showing splenic laceration. Contrast-enhanced CT abdomen (axial view, portal venous phase; slice thickness 5 mm; window/level 350/50 HU) showing low-attenuation perisplenic fluid collections and dependent hemoperitoneum (blue arrows) consistent with splenic laceration (AAST grade IV). The liver (L), spleen (S), and kidneys (K) are shown for anatomical orientation. AAST: American Association for the Surgery of Trauma; HU: Hounsfield unit

CT of the abdomen showed a high-grade splenic laceration (greater than American Association for the Surgery of Trauma {AAST} grade IV) with moderate hemoperitoneum and otherwise normal abdominal organs (Figure 3). Subtle periportal edema and mesenteric vascular engorgement were noted, representing minor components of the hypoperfusion shock complex, although other classic findings (adrenal or bowel wall hyperenhancement) were absent.

The patient underwent urgent exploratory laparotomy with splenectomy and drainage of hemoperitoneum. A thyroid profile was performed after the CT findings to rule out direct thyroid injury, and it was within normal limits. The patient also sustained a left femur fracture, for which a splint was initially applied, followed by internal fixation at a later stage. He gradually improved with supportive and surgical management and was subsequently discharged in stable condition. Follow-up CT imaging was not performed, as the patient exhibited complete clinical recovery with normalization of thyroid function, and further imaging was deemed unnecessary.

## Discussion

The finding of thyroid and perithyroidal edema with fluid in the setting of trauma is rare, with few cases reported in the literature [1-4]. It is generally considered part of the hypovolemic shock complex rather than a manifestation of direct thyroid trauma [1,4]. The radiological appearance typically includes diffuse thyroid enlargement, heterogeneous enhancement, and perithyroidal fluid without disruption of the gland capsule [2-4].

In contrast, direct thyroid injury from blunt neck trauma, such as rupture, hematoma, or parenchymal laceration, is extremely uncommon [5-7]. These cases often present with marked neck swelling, pain, and airway deviation due to compressive hematoma and may require surgical exploration or hemithyroidectomy [5-7]. Most of these injuries occur in previously normal thyroid glands following high-impact deceleration injuries or in patients with preexisting thyroid nodules or goiter [5-7].

The pathophysiology of shock-related thyroid changes remain uncertain. Several hypotheses have been proposed, including third-spacing of resuscitative fluids similar to periportal edema in trauma patients, hypoperfusion-induced interstitial edema due to severe hypovolemia, or a transient thyroidal hyperdynamic response attempting to maintain cardiac output in shock states [1-4]. The latter mechanism may explain the imaging appearance of a diffusely hyperenhancing thyroid gland without biochemical thyrotoxicosis [1,2,4].

On imaging, shock-related thyroid changes are characterized by symmetric low-density perithyroidal fluid and heterogeneous thyroid enhancement with preserved contour, distinguishing it from direct traumatic injury, which typically shows capsular disruption, hematoma, or tracheal deviation [1-4,5-7]. Recognition of this pattern in the appropriate clinical context, particularly in polytrauma patients, can prevent diagnostic confusion and avoid unnecessary interventions. As demonstrated in the present case and described by Lemke et al., the finding resolved with hemodynamic stabilization, confirming its transient and reversible nature.

## Conclusions

Shock-related thyroid changes are a rare but important radiological finding that occurs in the setting of profound hypovolemia and trauma. Recognizing its distinct CT features, heterogeneous thyroid enhancement with perithyroidal edema, is crucial to avoid misdiagnosis as direct thyroid injury or vascular trauma. This condition is typically self-limiting and does not require thyroid-specific intervention. Awareness of shock-related thyroid changes can therefore help clinicians and radiologists prevent unnecessary investigations or procedures and focus management on the underlying cause of hemorrhagic shock. As this represents a single-case observation, further studies are required to validate the proposed mechanisms.

## References

[1] Brochert A, Rafoth JB (2006). Shock thyroid: a new manifestation of the hypovolemic shock complex in trauma patients. J Comput Assist Tomogr.

[2] Kim WH, Kim MS, Kim JH, Lee KH, Lee JH (2021). Shock thyroid in a patient with septic shock: a case report and literature review. J Korean Soc Radiol.

[3] Han DH, Ha EJ, Sun JS, Jung SL (2017). Remarkable CT features of shock thyroid in traumatic and non-traumatic patients. Emerg Radiol.

[4] Lemke J, Schreiber MN, Henne-Bruns D, Cammerer G, Hillenbrand A (2017). Thyroid gland hemorrhage after blunt neck trauma: case report and review of the literature. BMC Surg.

[5] Perdon-Chan L, Roasa F (2022). Thyroid rupture secondary to blunt neck trauma: a case report. Craniomaxillofacial Res Innov.

[6] Nguyen CT, Bunevich J (2019). Hemorrhagic thyroid nodule resulting in expanding neck hematoma following blunt cervical trauma. Ear Nose Throat J.

[7] Zawawi F, Varshney R, Payne RJ, Manoukian JJ (2013). Thyroid gland rupture: a rare finding after a blunt neck trauma. Int J Pediatr Otorhinolaryngol.

